# PERSPECTIVE-TAKING DECLINE IN OLDER ADULTS WITH AND WITHOUT SUBJECTIVE COGNITIVE DECLINE

**DOI:** 10.1093/geroni/igad104.1541

**Published:** 2023-12-21

**Authors:** W Quin Yow, Nina Ye, Xiaoqian Li

**Affiliations:** Singapore University of Technology and Design, Singapore, Singapore; Singapore University of Technology and Design, Singapore, Singapore; Singapore University of Technology and Design, Singapore, Singapore

## Abstract

Subjective cognitive decline (SCD), a self-reported decline in cognitive function without objective cognitive decline, is an early risk marker of pathological aging (Jessen et al., 2020). Healthy older adults with SCD(+) were found to have lower objective cognition and increased rates of cognitive decline compared to those without SCD(-) (Morrison & Oliver, 2023). Cognitively-healthy older adults (OA) had shown a decline in perspective-taking abilities compared to younger adults (YA). However, little research has examined whether self-report SCD plays a role in such age-related declines. We examined age-related changes in perspective-taking abilities in OA with and without SCD vis-à-vis YA. Participants were 25 YA (Mage=22.44, range=20-27), 27 SCD- (Mage=68.67, range=60-77), and 20 SCD+ (Mage=66.55, range=60-72). OA were categorized as SCD-/SCD+ based on SCD-Questionnaire (Rami, 2014). SCD- and SCD+ groups were comparable in general cognition (Montreal Cognitive Assessment; p=.91). Participants completed the “owl” task assessing visual perspective-taking (VPT) and the “director” task assessing cognitive perspective-taking (CPT). Kruskal-Wallis tests revealed that YA outperformed OA (Zs>3.52, ps<.001) in VPT, with no significant difference between SCD- and SCD+ (Z=1.27, p=.21). Surprisingly, both YA and SCD+ were better than SCD- in CPT (Zs>2.34, ps<.04), and SCD+ did not differ significantly from YA (Z=0.02, p=.99). Our results suggest that while visual perspective-taking may decline with age regardless of SCD, cognitive perspective-taking may be preserved in older adults with SCD. SCD(+) may signal a hyperawareness of cognitive states in self and others, which in turn stimulate better cognitive perspective-taking. Implications of SCD on social cognition will be discussed.

